# Multi-level dilated residual network for biomedical image segmentation

**DOI:** 10.1038/s41598-021-93169-w

**Published:** 2021-07-08

**Authors:** Naga Raju Gudhe, Hamid Behravan, Mazen Sudah, Hidemi Okuma, Ritva Vanninen, Veli-Matti Kosma, Arto Mannermaa

**Affiliations:** 1 Institute of Clinical Medicine, Pathology and Forensic Medicine, Translational Cancer Research Area, University of Eastern Finland, P.O. Box 1627, 70211 Kuopio, Finland; 2 Department of Clinical Radiology, Kuopio University Hospital, P.O. Box 100, 70029 Kuopio, Finland; 3 Institute of Clinical Medicine, Radiology, Translational Cancer Research Area, University of Eastern Finland, P.O. Box 1627, 70211 Kuopio, Finland; 4 Biobank of Eastern Finland, Kuopio University Hospital, Kuopio, Finland

**Keywords:** Computational models, Image processing, Machine learning, Computational biology and bioinformatics

## Abstract

We propose a novel multi-level dilated residual neural network, an extension of the classical U-Net architecture, for biomedical image segmentation. U-Net is the most popular deep neural architecture for biomedical image segmentation, however, despite being state-of-the-art, the model has a few limitations. In this study, we suggest replacing convolutional blocks of the classical U-Net with multi-level dilated residual blocks, resulting in enhanced learning capability. We also propose to incorporate a non-linear multi-level residual blocks into skip connections to reduce the semantic gap and to restore the information lost when concatenating features from encoder to decoder units. We evaluate the proposed approach on five publicly available biomedical datasets with different imaging modalities, including electron microscopy, magnetic resonance imaging, histopathology, and dermoscopy, each with its own segmentation challenges. The proposed approach consistently outperforms the classical U-Net by 2%, 3%, 6%, 8%, and 14% relative improvements in dice coefficient, respectively for magnetic resonance imaging, dermoscopy, histopathology, cell nuclei microscopy, and electron microscopy modalities. The visual assessments of the segmentation results further show that the proposed approach is robust against outliers and preserves better continuity in boundaries compared to the classical U-Net and its variant, MultiResUNet.

## Introduction

Image segmentation is a classical computer vision problem aiming at extracting regions of interest (ROIs), which share specific and often similar characteristics. Semantic segmentation is an active area in the biomedical image segmentation tasks to identify pixels of organs or lesions from the background and links them to a class label. Biomedical image acquisition is prone to various limitations, such as low signal to noise ratio, motion artifacts, low spatial, and temporal resolution^1^, which impose challenges to properly segment the ROIs. There is an increasing interest in developing computer-aided diagnosis models, which can perform segmentation on biomedical images without human interventions^2^.

Deep convolutional neural networks (CNNs) trained by backpropagation^3^ have been successfully used for the image segmentation. Long et.al., trained an end-to-end model based on CNNs for pixel-wise semantic segmentation and introduced a novel ‘skip’ connection for combining low-level with high-level features^4^. Badrinarayan et.al., introduced a deep convolutional encoder-decoder architecture, consisting of convolutional layers (encoder) and de-convolutional layers (decoder) followed by a pixel-wise classifier, for a semantic segmentation task^5^. Ronneberger et.al., further extended^4^ and proposed the classical U-Net architecture, which can be trained end-to-end with fewer training examples^6^. The U-Net architecture is state-of-the-art and to date, different variants of the classical U-Net have been proposed for the biomedical image segmentation tasks^1,2,7–10^. Despite being successful, U-Net has some limitations, including loss of spatial information^7,9,10^ and difficulty in handling images with variations in lesion or tumor size^10^.

**Figure 1:**
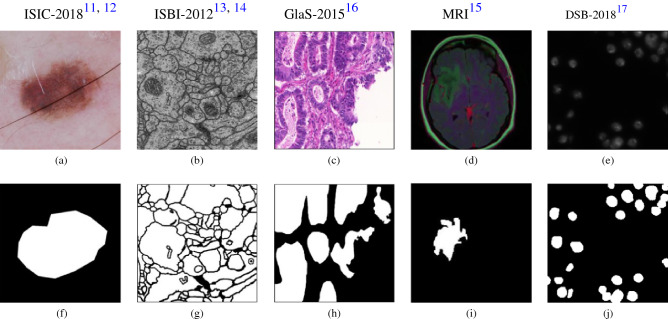
Example images from each publicly available biomedical imaging dataset used in this study. From left to right, the first row shows the images from **(a)** ISIC-2018 dermoscopy^11,12^, **(b)** ISBI-2012^13,14^ electron microscopy, **(c)** GlaS-2015^16^ histopathology, **(d)** MRI^15^, and **(e)** DSB-2018^17^ cell nuclei microscopy; the second row **(f–j)** shows their corresponding segmentation mask used as targets to train the segmentation models.

In this study, we propose a multi-level dilated residual network based on the classical U-Net architecture to address the U-Net limitations in several biomedical imaging datasets. We propose to replace convolutional blocks of the classical U-Net with the multi-level dilated residual (MLDR) blocks. Furthermore, we modify the skip connections by suggesting multi-level residual (MLR) network prior to concatenating features from the encoder to the decoder. We demonstrate our approach on five publicly available biomedical images with different modalities, namely, dermoscopy^11,12^, electron microscopy^13,14^, MRI^15^, histopathology^16^, and cell nuclei imaging^17^. An example from each dataset with the corresponding segmented binary mask is shown in Fig. 1. We compare our proposed approach against the classical U-Net and its state-of-the-art variants, including UNet++^7,9^, ResDUnet^1^, and MultiResUNet^10^, in a biomedical image segmentation task.

## Methods

### Classical U-Net architecture

The classical U-Net is an encoder-decoder based convolutional architecture^6^. The encoding unit encodes the input image into feature maps with lower dimensionalities and the decoding unit performs up-convolutional operations to generate segmentation maps with the same dimensions as the input image. The encoder consists of a sequence of two 3 × 3 convolutional operations, denoted as convolutional block, followed by a 2 × 2 max-pooling operation with stride of 2. After each max-pooling layer, the number of filters in the convolutional layers is doubled with an initial kernel size of 32. This sequence is repeated four times in the classical U-Net. The decoder unit up-samples the feature map using a 2 × 2 transposed convolutional operation followed by a sequence of two 3 × 3 convolutional operations. Like the encoder, the up-sampling and the two convolutional operations are repeated four times in the decoder, each time halving the number of kernels. Finally, the segmentation mask is generated by a 1 × 1 convolutional layer.

### Multi-level dilated residual convolutions

The convolution operation is powerful and capable of extracting features automatically by sliding the kernel (filter) over the input image. The appreciable property of convolutions is that they are translationally equivariant, meaning that a small amount of shift in an input image, the output remains the same, shifted by the same amount^18^. U-Net encoder-decoder based architecture incorporates convolutional layers to extract more robust high-level semantic features. The output (feature maps) of convolutional layers are down-sampled using max-pooling layers, then are restored back to the original size using up-sampling or deconvolution operation. However, after the pooling operation, the translational equivariant property may not hold, making the network sensitive to small shifts in an input image^18,19^.

The regions of interest of biomedical images are irregular and have different scales (see some examples in Fig. 1). Therefore, it is required to develop an architecture to be robust to analyze ROIs at different scales and variations. The classical U-Net has limitation to handle such variations for predicting the true segmentation^10^. Different variants of the classical U-Net have been already proposed to overcome such limitations^1,2,7–10^. In^10^, Ibtehaz et al., replaced the convolutional blocks of the classical U-Net with inception-like blocks^20^ using residual shortcut connections^21^ to address the variation of scales in the images. Yu et al., showed that dilated convolutions increase the effective receptive field size, thus, more spatial information at different scales could be extracted^22^. Deep residual neural networks followed by the sequence of batch normalization (BN), rectified linear unit (ReLU), and convolution operation (in short, BN-ReLU-Conv) were suggested to alleviate the vanishing gradient problem, to improve the performance of deep neural networks^23^. Zhang et al., suggested that multiple levels of residual networks, i.e. residual-of-residual connections, promote the learning capability of the residual connections and could overcome the overfitting problem^24^.

**Figure 2:**
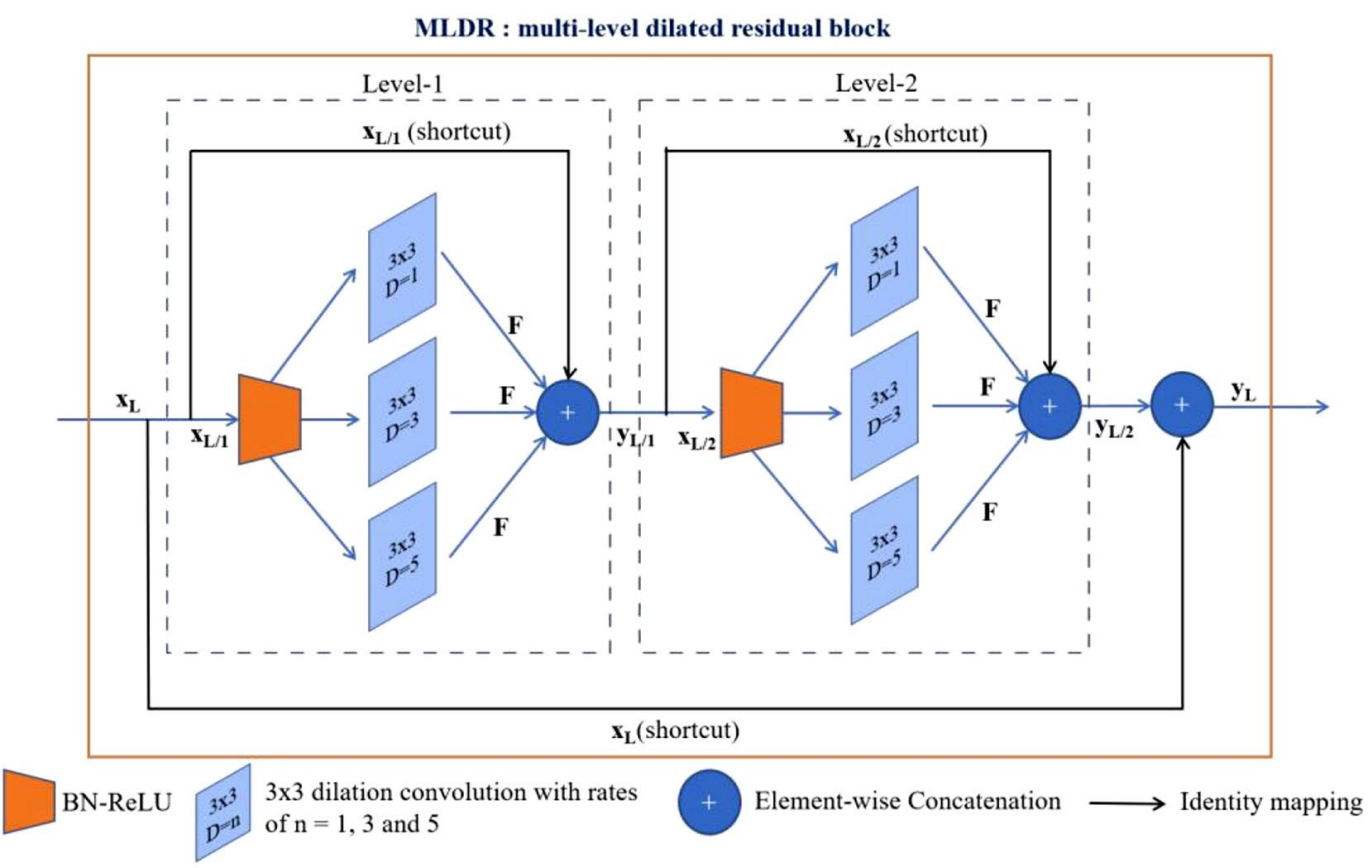
A schematic representation of the MLDR block. In this study, we suggest replacing the convolutional block in the classical U-Net^6^ with the MLDR block. Each MLDR block consists of two levels, each having a sequence of BN-ReLU followed by three 3 × 3 parallel dilated convolutions at dilation rates of 1, 3, and 5 with the residual connection, to extract features at different resolutions.

In this study, for the first time, we are introducing to use the multi-level dilated residual convolutions for the semantic segmentation of the biomedical images. Each level (denoted as L/N) of a multi-level residual of residual connection is expressed as follows^24^:

$${y}_{L/N}=h\left({x}_{L/N}\right)+F\left({x}_{L/N},{W}_{L/N}\right),$$

1 $${x}_{L/N+1}=\,\,f\left({y}_{L/N}\right)$$

where ${x}_{L/N}$ and ${x}_{L/N+1}$ are the input and the output of the *L*th block, respectively. $h\left({x}_{L/N}\right)=\,\,{x}_{L/N}$ is an identity mapping, $F$ is a residual mapping function with weights ${W}_{L/N}$, and $f$ is a ReLU function. We suggest replacing $F$ with dilated convolutions at rate $d$, expressed as follows^22^:

$$\left({x}_{L/N\mathrm{*}d}{W}_{L/N}\right)\left(s\right)={\sum\limits }_{s\,\,+\,\,dt}{x}_{L/N}\left(s\right){W}_{L/N}\left(t\right),$$

2 $$F\left({x}_{L/N},{W}_{L/N}\right)\,\,=\,\,\left({x}_{L/N\mathrm{*}d}{W}_{L/N}\right)\left(s\right)\,\,$$

where $\mathrm{*}d$ is the dilated convolution operation. In this study, we suggest replacing the convolutional block of the classical U-Net with the two-level (N = 2) dilated residual convolutions of rates $d\,\,=\,\,\mathrm{1,3},\,\,and\,\,5$, denoted as MLDR block. Each level of the MLDR block consists of a sequence of BN-ReLU followed by three 3 × 3 parallel convolutions at dilation rates of 1, 3, and 5 with the residual connection, to extract features from the biomedical images with different sizes and scales (Fig. 2).

### Skip connections with multi-level residual block

The classical U-Net architecture introduced skip connections to improve the segmentation accuracy^6^. The skip connections combine the low-level features, extracted from the encoder unit, with the high-level features of the corresponding decoder unit to recover the spatial information lost during the max-pooling operation^6^. Despite preserving the spatial information of the target mask, most of the fine-grained details are lost and thus, adversely affecting the predicted segmentation^10^. Zhou et.al., re-designed the skip connections by introducing a series of nested dense convolutional blocks to reduce the semantic gap between the features of the encoder and the decoder prior to the fusion^7,9^. Ibtehaz et.al., further incorporated convolutional layers with residual connections into the skip connections^10^.

**Figure 3:**
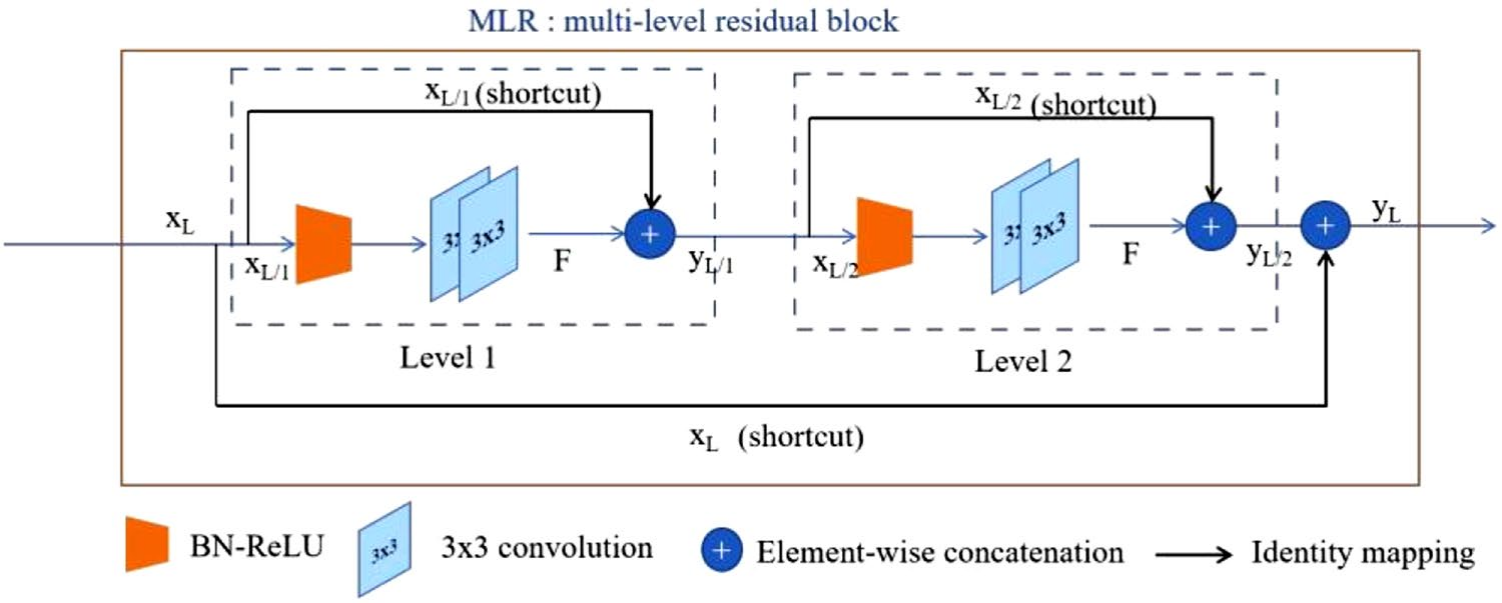
In this study, we propose to incorporate the residual-of-residual connection^24^ as non-linear skip connection prior to combining features extracted from the encoder to the decoder. We denote these non-linear layers as the MLR block. The MLR block contains two levels, each having a sequence of BN-ReLU followed by two 3 × 3 standard convolutions with a residual connection.

Inspired by^10,24^, we propose to use non-linear layers as skip connections, which consist of multi-level residual (MLR) block, resembling the two levels of the residual-of-residual connection. Incorporating the MLR block into the skip connections restores the spatial and temporal information loss and enhances the network learning capability to accurately segment the ROIs. The MLR block (Fig. 3) contains two levels, each having a sequence of BN-ReLU followed by two 3 × 3 standard convolutions (*d* = 1 in Eq. 2) with a residual connection.

### Multi-level dilated residual network (MILDNet)

**Figure 4:**
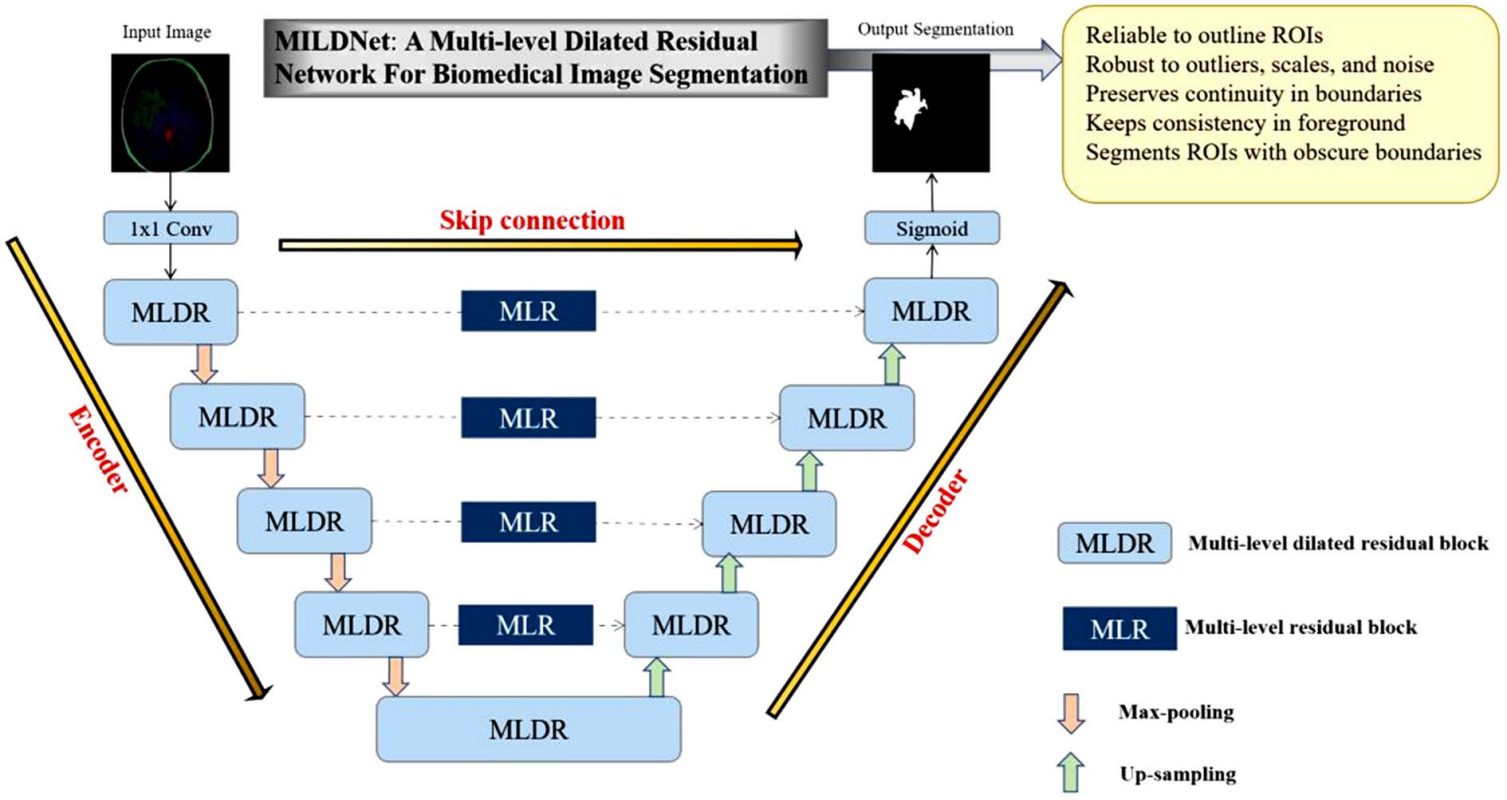
Schematic diagram of the proposed approach for the biomedical image segmentation task. Unlike the convolutional blocks in the classical U-Net, we propose to incorporate the MLDR blocks to overcome some of the classical U-Net limitations, including the difficulty in handling images with variations in tumor sizes and scales. We also propose to use the MLR blocks into the skip connections, as non-linear layers, to further enhance restoring the spatial information, which is usually lost in the classical U-Net.

Figure 4 illustrates an overall overview of our proposed approach. Similar to the classical U-Net, the MLDR block in the encoder unit is followed by a 2 × 2 max-pooling operation with stride of size 2 to reduce the dimensions of the extracted feature maps to half. With the increase in the depth of the architecture, the kernel size of the convolution operation is double with the initial kernel size of 32. In the decoder unit, 2×2 transpose convolutions up-sample the input features followed by the MLDR block. The final prediction layer is a 1×1 convolution operation activated with sigmoid function to predict the segmentation mask of the given input image.

## Experimental setup

### Datasets

In this study, we evaluate the performance of the proposed and the baseline models on five biomedical datasets of different imaging modalities, including dermoscopy, electron microscopy, MRI, histopathology, and cell nuclei microscopy. Table 1 summarizes each dataset, provides the extraction protocol, and the annotation details of each image modality. Note that we do not have control over the quality of the ground truths (annotations) and they are already provided with the biomedical images in each dataset.

**Table 1:** Five publicly available biomedical imaging datasets used in this study for the semantic segmentation. Note that the images are available with varying sizes within some datasets.

Dataset	Modality	No. of images	Majority im age size	Description
ISIC-2018^11,12^	Dermoscopy	2594	1022 × 767	Dermoscopy is an imaging technique that eliminates the skin surface reflection to enhance visualization of the deeper skin layers. We have acquired the dermoscopy images from the ISIC-2018; skin lesion analysis towards melanoma detection challenge.
ISBI-2012^13,14^	Electron microscopy	30	512 × 512	This dataset contains a serial section transmission electron microscopy of the drosophila first instar larva ventral nerve cord. The dataset is provided by the ISBI-2012; 2D electron microscopy segmentation challenge.
MRI^15^	MRI	1144	256 × 256	This dataset contains brain MRI images and segmentation masks created by manual fluid-attenuated inversion recovery acquired from 110 patients included in the cancer genome atlas lower-grade collection.
GlaS-2015^16^	Histopathology	165	775 × 522	This dataset acquired from the gland segmentation in colon histology image challenge. The images are scanned whole slide histology images of the colon, in which epithelial glands are annotated.
DSB-2018^17^	Cell nuclei mi croscopy	670	320 × 256	This dataset contains segmented nuclei images acquired under different conditions by changing the cell type, magnification, and imaging modality (bright-field vs. fluorescence).

### Baseline models for performance comparison

**Figure 5:**
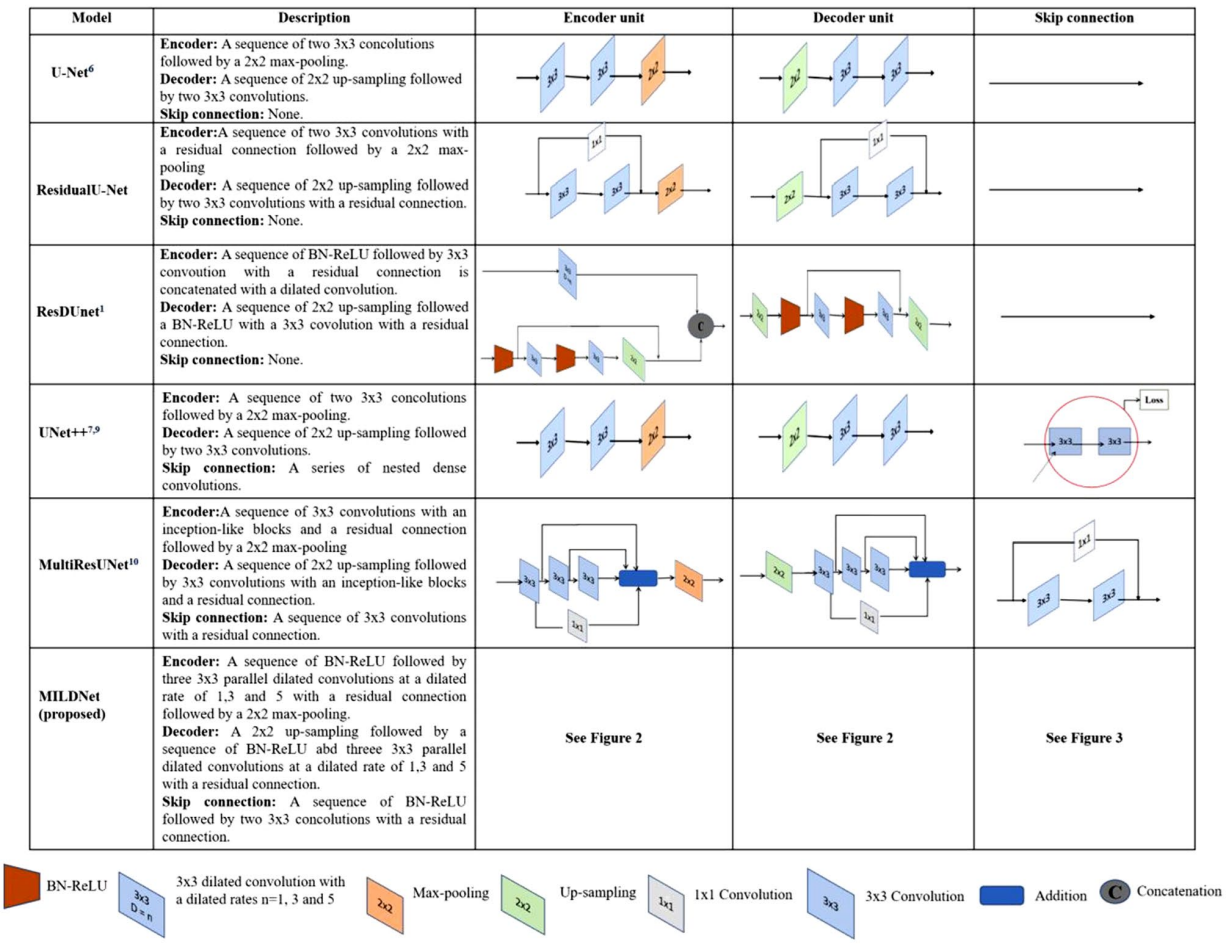
The differences in the architectures of the proposed MILDNet and the baseline approaches, including the classical U-Net, ResidualU-Net, ResDUnet, UNet +  +, and MultiResUNet. For visual comparison, we have recreated the encoder, decoder, and skip connection structures of the baseline approaches following the network configuration represented in their original studies.

For comparison purposes, we adopted the classical U-Net as well as a number of recently proposed extensions of the classical U-Net architecture^6^, including the UNet++^7,9^, ResDUnet^1^, and the MultiResUNet^10^. We also incorporated a residual shortcut connection in the convolutional block of the classical U-Net to develop a residual-based U-Net architecture, denoted as ResidualU-Net, and used it as one of the baseline approaches. The main differences between the proposed architecture and the baseline approaches are illustrated in Fig. 5.

We obtained the source code of the classical U-Net from^25^, following the network configuration represented in the original U-Net paper. The UNet++ and the MultiResUNet were originally implemented in the Keras framework, respectively in^9^ and^10^, and we re-implemented them in the Pytorch 1.3.1 framework. We also implemented ResDUnet in Pytorch following the network architecture proposed in the original paper^1^. The models were trained using a machine equipped with Nvidia Tesla V100 16 GB graphic card on Intel Xeon Processor provided by the IT service Center for Science (CSC) Finland^26^.

### Training protocol

We generated image patches of size 256* 256 with padding of 16 for the ISBI-2012 and GlaS-2015 datasets (due to fewer number of training data) to increase the number of data samples. We used Patchify^27^, a python-based library, to generate image patches of size 256* 256 with a padding of 16 to increase the number of data samples. For ISBI-2012 dataset, we generated 4 patches from each image, resulting in a sample size of 120 images, in total. Similarly, from GlaS-2015 dataset, we generated 11 patches from each image, resulting in a sample size of 1815 images.

Additionally, we applied affine, elastic, and pixel-level data augmentation techniques using Albumentations Python library^28^ during the training process. Data augmentation was shown to help generalization capability of the neural networks and to avoid over-fitting problem in previous studies^29,30^. Affine transformations include rotations (0^0^, 60^0^, 120^0^ , 180^0^ , 270^0^ ), horizontal and vertical flipping, random scaling (scale limit = 0.1, interpolation = 1), and random shear (limit = [− 45, 45]). We noticed that the affine transformations had less or no impact on improving the segmentation accuracy. Thus, we also included elastic deformation transformations from^31^ to introduce shape variations and pixel-level transformations to vary pixel-level intensity. Transformations include ColorJitter (brightness = 0.2, contrast = 0.2, saturation = 0.2, hue = 0.2), GaussianBlur (blur_limit = (3, 7), sigma_limit = 0), and GaussNoise (var_limit = (10.0, 50.0), mean = 0) (See the supplementary file for further details).

Each dataset is split into 70% for training (training set) and 30% for the performance evaluation (test set). The training set is used to train and fine-tune the models using a 5-fold cross validation (CV) for 100 epochs. The test set is used to evaluate each model against the training folds and then, the mean value is computed as the final prediction performance for each model. Table 2 illustrates the dataset splitting protocol for each dataset.

**Table 2:** The dataset splitting protocol followed in this study.

Dataset		ISIC-2018	ISBI-2012	MRI	GlaS-2015	DSB-2018
Training folds	Fold 1	363	16	160	253	94
	Fold 2	363	16	160	253	94
	Fold 3	363	16	160	253	94
	Fold 4	363	16	160	253	94
Validation fold	Fold 5	364	20	161	253	93
Test set		778	36	343	550	201

Each dataset is first partitioned into the training and the test sets. The training set is further split in a 5-fold CV, where 4-folds are used for training and the last fold for validation. The test set is used to evaluate each model against the 5-folds and then, the mean value is computed as the final segmentation performance of each model. For ISBI-2012 and GlaS-2015 datasets, we have used patch-wise training to increase the number of data samples.

The dimensions of all input medical images are resized to 256* 256 with bilinear interpolation and normalized to the range [0, 1] using a min-max scaler^32^. In this study, we considered each model architecture to have a depth of 5 with an initial kernel size of 32. With the increase in depth, the kernel size is multiplied by a factor of 2.

For model interpretation, we used gradient saliency maps^33^. Saliency maps are generated as the derivative of the model output with respect to the input features to visualize regions within an input image, which contribute the most to the corresponding output. For a given input image, we computed saliency maps for each decoder layer, and then combined them by averaging over all the saliency maps to form a single saliency map. We up-sampled the saliency maps to match the dimension of the input images.

### Loss function

Binary cross-entropy with logits^34^ is used to measure the loss between the actual and the predicted segmentation masks.

For a pixel index $i$ and an input image $X\,\,=\,\,{\left\{x\right.}_{i}\,\,\in \,\,R\,\,|\,\,i\,\,=\,\,0,\,\,1,\,\,\dots ,\,\,\left.255\right\},$ Let $Y\,\,=\,\,{\left\{y\right.}_{i}=0\,\,or\,\,1\,\,|\,\,i\,\,=\,\,0,\,\,1,\,\,\dots ,\,\,\left.255\right\}$ and $\widehat{Y}\,\,=\,\,{\left\{\widehat{y}\right.}_{i}\in \,\,[\mathrm{0,1}]\,\,|\,\,i\,\,=\,\,0,\,\,1,\,\,\dots ,\,\,\left.255\right\}$ be the ground-truth and predicted segmentation masks, respectively. Then, the binary cross-entropy is defined as^34^:

3 $$L\left(X,Y,\widehat{Y}\right)=\sum _{{x}_{i}\,\,\in \mathrm{X}}(-{y}_{i}\,\,\mathrm{log}(\widehat{{y}_{i}})\,\,+\,\,(1-{y}_{i})\,\,\mathrm{log}(1-\widehat{{y}_{i}}))$$

### Evaluation metrics

We selected the widely used dice coefficient (DC), intersection over union (IoU), and Hausdorff distance (HD) for the quantitative analysis of the segmentation results. These metrics are defined as follows^35^:

4 $$DC\,\,=\,\,\frac{2\,\times\,\,\left(Y\cap \widehat{Y}\right)}{\left|Y\right|\,\,+\,\,\left|\widehat{Y}\right|}$$

5 $$IoU\,\,=\,\,\frac{Y\,\,\cap \,\,\widehat{Y}}{Y\,\,\cup \,\,\widehat{Y}}$$

6 $$HD=max\left(h\left(Y,\widehat{Y}\right),h\left(\widehat{Y},Y\right)\right),$$

where, $\left|.\right|$ denotes absolute values,

7 $$h(Y,\,\,\widehat{Y})\,\,=\,\,\,\,{max}_{{y}_{i}\,\,\in \,\,Y}\,\,({min}_{{\widehat{y}}_{i}\,\,\in \,\,\widehat{Y}}\,\,(d({y}_{i},\,\,{\widehat{y}}_{i})),$$

and

8 $$h(\widehat{Y},\,\,\,\,Y)\,\,={max}_{{\widehat{y}}_{i}\,\,\in \,\,\widehat{Y}}\,\,({min}_{{y}_{i}\,\,\in \,\,Y}\,\,(d(\,\,{\widehat{y}}_{i},\,\,{y}_{i})),$$

$d\left({y}_{i},{\widehat{y}}_{i}\right)$ and $d\left({\widehat{y}}_{i},{y}_{i}\right)$ denote the Euclidean distance between ${y}_{i}$ and ${\widehat{y}}_{i}$; $h\left(Y,\widehat{Y}\right)$ measures the directed HD from $Y\,\,to\,\,{\widehat{Y}}$ by computing the minimum distance from ${y}_{i}$ to its nearest neighbor in ${\widehat{Y}}$ and then, the maximum distance is considered as the HD value between $Y\,\,and\,\,\widehat{Y}$. Similarly $h\left(\widehat{Y},\,\,\,\,Y\right)$ measures the directed HD from $\widehat{Y}$ to $Y$ by computing the minimum distance from ${\widehat{y}}_{i}$ to its nearest neighbor in $Y$ and then, the maximum distance is considered as the HD value between $\widehat{Y}\,\,and\,\,Y$. Finally, the degree of mismatch between $Y\,\,and\,\,\widehat{Y}$ is computed as the maximum HD value between $h\left(Y,\widehat{Y}\right)$ and $h\left(\widehat{Y},\,\,\,\,Y\right).$

## Results and discussion

### Finding optimal hyper-parameters using grid-search

We first performed a grid-search^36^ over the model hyper-parameters, including batch size, training optimizer, momentum, and learning rate scheduler; and the network architecture hyper-parameters, including depth, levels, and dilation rates, to find the optimal values for the proposed approach. The combination of the hyper-parameters in the grid-search is presented in Table 3. We found that the batch size of 4, the Adam optimizer, the momentum of 0.9, and the reduced learning rate on plateau (ReduceLROnPleateau) with an initial learning rate of 0.001; and the network architecture of depth 5, level 2, and dilation rates of [1, 3, 5] show a consistent accuracy within each model and the imaging modalities. The optimal values are then used to train the models for each dataset in a 5-fold CV and the test sets are used to evaluate the models against each fold. We initialized the convolutional layers with Xavier initialization^37^.

**Table 3:** Combination of the hyper-parameter settings and their optimal values found using grid-search in a 5-fold CV.

Hyper-parameters	Grid-search values		Optimal values of MILDNet
Batch size	[4, 8, 16, 32, 64]		4
Training optimizers^38^	[Stochastic gradient descent, Adam, RMSprop]		Adam
Learning rate schedulers^39^	[StepLR, MultiStepLR, CosineAnnealingLR, ReduceLROnPlateau, CyclicLR]		ReduceLROnPlateau
Learning rate	[le–2, le−3, le−4, le−5]		le−3
Momentum	[0.3, 0.6, 0.9]		0.9
Depth	[3, 4, 5, 6]		5
Dilation rates	[{1, 2, 6}, {1, 2, 5}, {1, 2, 4}, {1, 3, 5}, {2, 4, 8}, {1, 3, 7}]		{1, 3, 5}
Levels	[1, 2, 3, 4, 5]		2

In this study, we used the optimal hyper-parameter values of the MILDNet to train the baseline approaches. The same folds are also used during training, validation, and testing of the proposed and the baseline approaches.

### Residual-of-residual skip connections (MLR blocks) improve the segmentation accuracy

To evaluate the impact of the MLR skip connection on the segmentation accuracy, we trained the proposed approach with and without inclusion of the MLR blocks into the skip connections (prior to concatenating the features from the encoder unit to the corresponding decoder unit) using the optimal hyper-parameters and the validation sets in 100 epochs.

**Figure 6:**
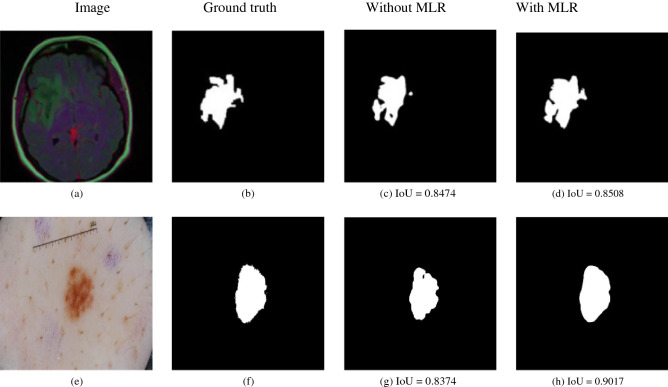
Two visual examples from the MRI^15^ **(a)** and the dermoscopy^11,12^ **(e)** images; and their corresponding ground truth masks **(b,f)** showing that the presence of the MLR blocks in the skip connections of the MILDNet enhances the segmentation accuracy, with the ground truths given in 6b and 6f. The predicted masks for the skip connections with the MLR blocks **(d,h)** preserved the continuity in the boundaries. The skip connections without the MLR blocks **(c,g)** resulted in the loss of some valuable information about the boundaries and the ROI shape.

Table 4 shows that using the MLR blocks in the MILDNet (without data augmentation) slightly improves the segmentation accuracy by on average 2% relative improvement in terms of DC, considering all the datasets. Similar performance gain is also observed, when including the MLR blocks in the baseline U-Net. Figure 6 illustrates that the predicted segmentation masks are visually more similar to the gourd-truth binary masks (Fig. 6b) and (Fig. 6f), especially in preserving the shape and the continuity in boundaries, when using the MLR blocks in the MILDNet (Fig. 6d,h) over the direct skip connections without inclusion of the MLR blocks (Fig. 6c,g) in the MRI (Fig. 6a) and dermoscopy (Fig. 6e) images. A remarkable segmentation improvement is observed in the dermoscopy example with IoU = 0.9017 using the MLR blocks (Fig. 6h) compared to IoU = 0.8374 without using the MLR blocks (Fig. 6g).

**Table 4:** The impact of the residual-of-residual skip connections (MLR blocks) on the segmentation accuracy using the validation sets. ↑: The higher value is better; ↓: The lower value is better.

Dataset	Models	DC ↑	IoU ↑	HD ↓
ISBI-2012 electron microscopy	U-Net (baseline) U-Net (with MLR) MILDNet (without MLR) MILDNet (with MLR)	0.84 ± 0.0004 0.86 ± 0.0004 0.92 ± 0.0005 **0.96 ± 0.0005**	0.79 ± 0.0004 0.80 ± 0.0005 0.90 ± 0.0005 **0.92 ± 0.0005**	9.730 ± 0.0022 9.654 ± 0.0022 9.481 ± 0.0023 **9.395 ± 0.0022**
ISIC-2018 dermoscopy	U-Net (baseline) U-Net (with MLR) MILDNet (without MLR) MILDNet (with MLR)	0.91 ± 0.0007 0.92 ± 0.0007 0.94 ± 0.0005 **0.94 ± 0.0005**	0.87 ± 0.0011 0.88 ± 0.0011 0.89 ± 0.0001 **0.90 ± 0.0001**	15.962 ± 0.014 15.341 ± 0.014 7.96 ± 0.0018 **7.54 ± 0.0018**
MRI	U-Net (baseline) U-Net (with MLR) MILDNet (without MLR) MILDNet (with MLR)	0.86 ± 0.0003 0.86 ± 0.0003 0.87 ± 0.0003 **0.88 ± 0.0003**	0.77 ± 0.0003 0.79 ± 0.0003 0.80 ± 0.0002 **0.81 ± 0.0002**	14.98 ± 0.0027 14.128 ± .0027 13.824 ± 0.0020 **13.62 ± 0.0020**
GlaS-2015 histopathology	U-Net (baseline) U-Net (with MLR) MILDNet (without MLR) MILDNet (with MLR)	0.82 ± 0.0004 0.84 ± 0.0004 0.85 ± 0.0003 **0.87 ± 0.0003**	0.70 ± 0.0003 0.74 ± 0.0003 0.77 ± 0.0004 **0.78 ± 0.0002**	16.0 ± 0.0027 16.0 ± 0.0027 **15.52 ± 0.0022** 15.606 ± 0.0020
DSB-2018 cell nuclei microscopy	U-Net (baseline) U-Net (with MLR) MILDNet (without MLR) MILDNet (with MLR)	0.88 ± 0.0005 0.90 ± 0.0005 0.92 ± 0.0003 **0.95 ± 0.0003**	0.79 ± 0.0004 0.83 ± 0.0004 0.89 ± 0.0002 **0.90 ± 0.0002**	4.258 ± 0.0022 4.224 ± 0.0022 4.129 ± 0.0020 **4.078 ± 0.0020**

The results suggest that the presence of the MLR blocks in the skip connections improves preserving the spatial and contextual information, which is usually lost during the concatenation of the features from the encoder to the decoder units in the classical U-Net. Therefore, we incorporate the MLR blocks into the skip connections in the following experiments for the enhanced semantic segmentation.

### MILDNet outperforms the classical U-Net and other baselines in segmenting the biomedical images

Table 5 compares the segmentation accuracy of the MILDNet approach with and without data augmentation against the classical U-Net, the UNet++, the MultiResUNet, the ResDUnet, and the ResidualU-Net, using the test sets of the five biomedical datasets.

**Table 5:** MILDNet outperforms the classical U-Net and other baselines in segmenting the biomedical images using the test sets.

Dataset	Models	DC↑		IoU↑		HD↓		
ISBI-2012 electron microscopy	U-Net	0.84 ± 0.0004		0.79 ± 0.0005		9.730 ± 0.0022		
	UNet + +	0.84 ± 0.0004		0.88 ± 0.0007		9.685 ± 0.0022		
	ResidualU-Net	0.84 ± 0.0005		0.89 ± 0.0010		10.327 ± 0.0031		
	ResDUnet	0.89 ± 0.0002		0.80 ± 0.0004		10.289 ± 0.0020		
	MultiResUNet	0.88 ± 8.36		0.79 ± 9.085		9.88 ± 0.058		
	MILDNet (without augmentation)	0.96 ± 0.0005		0.92 ± 0.0005		9.395 ± 0.0031		
	MILDNet (with augmentation)	**0.98 ± 1.386**		**0.93 ± 1.252**		**9.254 ± 1.75**		
ISIC-2018 dermoscopy	U-Net	0.91 ± 0.0007		0.87 ± 0.0011		15.962 ± 0.014		
	UNet + +	0.93 ± 0.0005		0.88 ± 0.0009		8.798 ± 0.007		
	ResidualU-Net	0.92 ± 0.0007		0.87 ± 0.0011		15.720 ± 0.014		
	ResDUnet	0.93 ± 0.0006		0.88 ± 0.0011		15.962 ± 0.014		
	MultiResUNet	0.93 ± 0.0006		0.87 ± 0.0010		15.962 ± 0.014		
	MILDNet (without augmentation)	0.94 ± 0.0005		0.90 ± 0.0001		7.54 ± 0.004		
	MILDNet (with augmentation)	**0.94 ± 0.0042**		**0.91 ± 0.036**		**7.39 ± 0.064**		
MRI	U-Net	0.86 ± 0.0003		0.77 ± 0.0003		14.98 ± 0.0027		
	UNet + +	0.86 ± 0.0002		0.76 ± 0.0003		15.42 ± 0.0027		
	ResidualU-Net	0.83 ± 0.003		0.72 ± 0.0003		15.36 ± 0.0022		
	ResDUnet	0.85 ± 0.0003		0.76 ± 0.0004		15.99 ± 0.0024		
	MultiResUNet	0.85 ± 0.0003		0.78 ± 0.0004		15.53 ± 0.0022		
	MILDNet (without augmentation)	0.88 ± 0.0002		**0.81 ± 0.0002**		13.62 ± 0.0020		
	MILDNet (with augmentation)	**0.89 ± 0.005**		0.80 ± 0.003		**13.02 ± 0.0082**		
GlaS-2015 histopathology	U-Net	0.82 ± 0.0004		0.70 ± 0.0003		16.0 ± 0.0027		
	UNet + +	0.87 ± 0.0004		0.78 ± 0.0003		15.998 ± 0.0027		
	ResidualU-Net	0.85 ± 0.0003		0.75 ± 0.0002		15.963 ± 0.0020		
	ResDUnet	0.83 ± 0.0002		0.72 ± 0.0002		16.0 ± 0.0020		
	MultiResUNet	0.84 ± 0.0003		0.74 ± 0.0003		15.606 ± 0.0020		
	MILDNet (without augmentation)	**0.87 ± 0.0003**		0.78 ± 0.0002		15.836 ± 0.0027		
	MILDNet (with augmentation)	0.86 ± 0.032		**0.80 ± 1.294**		**15.408 ± 0.0574**		
DSB-2018 cell nuclei microscopy	U-Net	0.88 ± 0.0005		0.79 ± 0.0004		4.257 ± 0.0022		
	UNet + +	0.94 ± 0.0004		0.89 ± 0.0003		4.631 ± 0.0027		
	ResidualU-Net	0.92 ± 0.0004		0.86 ± 0.0003		4.194 ± 0.0027		
	ResDUnet	0.93 ± 0.003		0.87 ± 0.0003		4.339 ± 0.0027		
	MultiResUNet	0.94 ± 0.0004		0.88 ± 0.0004		4.423 ± 0.0022		
	MILDNet (without augmentation)	**0.95 ± 0.0003**		0.90 ± 0.0002		**4.078 ± 0.0020**		
	MILDNet (with augmentation)	0.94 ± 1.208		**0.91 ± 0.328**		4.264 ± 0.022		

For the MILDNet, we have also applied data augmentation techniques during training. The evaluation metrics are calculated from the network output without applying further post-processing on the predicted binary masks. ↑: The higher value is better;↓: The lower value is better.

MILDNet with data augmentation has resulted in slightly superior segmentation performance compared to MILDNet without data augmentation in all except the MRI dataset, in terms of IoU. For consistency, hereafter, we choose MILDNet without data augmentation to compare segmentation results and for visual assessment. MILDNet outperforms all the baselines in segmenting the biomedical images. In particular, MILDNet consistently outperforms the classical U-Net by relative improvements of 2%, 3%, 6%, 8%, and 14%, respectively for the MRI, the ISIC-2018 dermoscopy, the GlaS-2015 histopathology, the DSB-2018 cell nuclei microscopy, and the ISBI-2012 electron microscopy biomedical images, in terms of DC. Similar performance gain is also observed in IoU and HD metrics. MILDNet also outperforms the recently proposed MultiResUNet approach by relative improvements of 1%, 1%, 1%, 4%, and 4%, respectively for the ISIC-2018 dermoscopy, the DSB-2018 cell nuclei microscopy, the ISBI-2012 electron microscopy, the MRI, and the GlaS-2015 histopathology datasets, in terms of DC. Interestingly, the ResidualU-Net approach achieves higher segmentation accuracy over the classical U-Net in all, except the MRI dataset.

**Figure 7:**
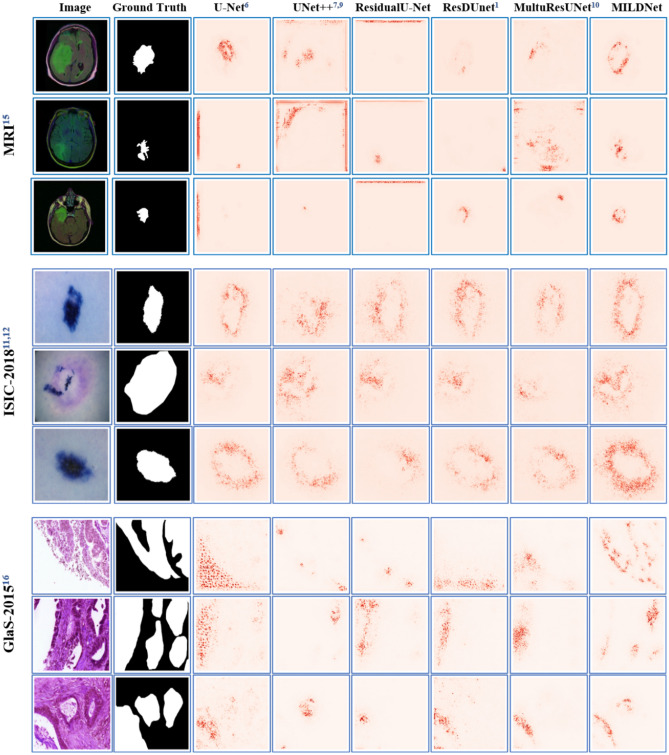
Saliency maps for the MRI, the dermoscopy, and the histopathology examples. Regions that have a high impact on the models’ final decision are highlighted.

Figure 7 illustrates the saliency maps of some examples from the MRI, the dermoscopy, and the histopathology datasets for all the models. From these examples, we can see that MILDNet concentrates much better on the ROIs in images with complex background as in the MRI and the histopathology datasets. For the dermoscopy images, which have better distinction between foreground and background, all models attend favorably to the ROIs.

Note that the variation observed in the relative changes from dataset to dataset may come from the segmentation challenges associated with each biomedical image modality. For example, in the ISBI-2012 electron microscopy dataset, the ROI covers the majority of the images, thus models may tend to oversegment the images. Illumination variation and different types of textures presented in the ISIC-2018 dermoscopy dataset make segmentation more difficult. For some images in the MRI dataset, it is difficult to visually identify tumors from the background due to vague ROI boundaries. In addition, brain tumors have different size, shape, and structure, which make the segmentation challenging. Similarly, irregular boundaries and structures separating the tumor and non-tumor regions in the histopathology images. In the cell nuclei microscopy dataset, some images contain bright objects, which resemble the cell nuclei (ground-truth) and may act as outliers in the segmentation. The visual assessments of the segmentation results will present some of these challenges in a later section.

We also noticed a difference between the segmentation IoU values of our proposed method with the IoU values reported in the literature. For example, the IoU values of U-Net and UNet++ for DSB-2018 in^7,9^ are 90.57 ± 1.26 and 92.44 ± 1.20, respectively, while in our study are 0.79 ± 0.0004 and 0.89 ± 0.0003. This variation is due to using different data-splitting protocol and the optimal hyper-parameters, and further we did not apply any post-processing techniques, such as watershed algorithm^40,41^, for separating the clustered nuclei.

**Figure 8:**
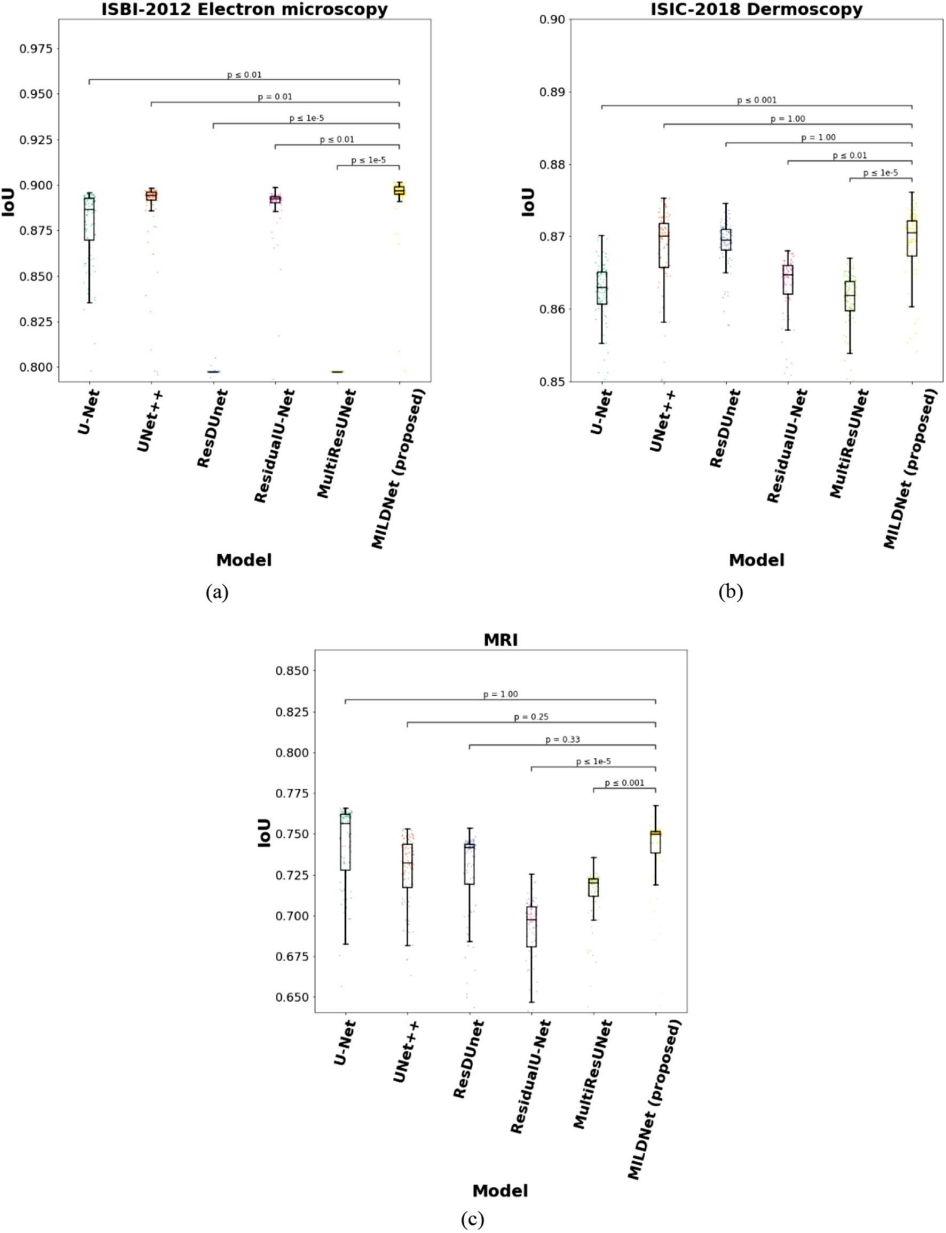

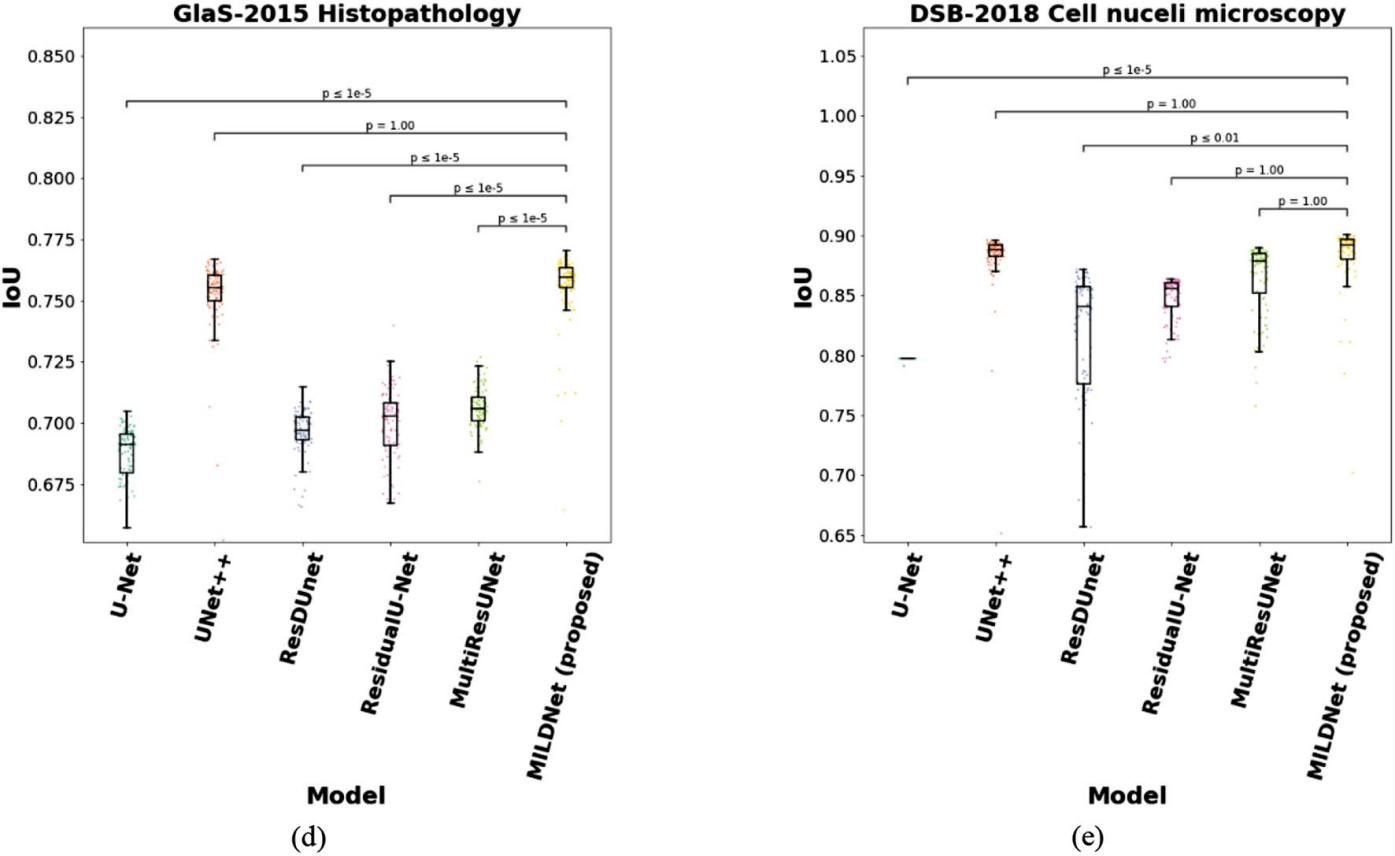
Statistical significance for the differences in segmentation performances of the MILDNet and the baseline approaches using *t*-test. The differences between the IoU values of the MILDNet and the baselines are statistically significant when *p*-value ≤ 0.05. Y-axis represents the overall IoU value of each model using a 5-fold CV on the entire dataset by merging the training, validation, and test sets of each biomedical dataset. The sub-figures **(a–e)** represents the box plot with the baseline approaches U-Net, UNet + + , ResDUnet, MultiResUNet and MILDNet (proposed) on the x-axis and the IoU values on the y-axis for all the five biomedical datasets used in this work.

Finally, we performed a 5-fold CV on the entire datasets by merging the training, validation, and test sets of each biomedical dataset, then, ran a simple analysis of statistical significance as *t*-test to check if the differences between the IoU values of the proposed and the baseline systems are statistically significant with *p*-value ≤ 0.05. The results in Fig. 8 show that the proposed MILDNet approach without data augmentation demonstrates a significant IoU improvements with *p*-value ≤ 0.05 over the classical U-Net in all except the MRI dataset, however, with a smaller standard deviation in this dataset. Similarly, the IoU differences between the MILDNet and the state-of-the-art MultiResUNet approach are statistically significant with *p*-value ≤ 0.05 in all except the DSB-2018 cell nuclei microscopy dataset.

### Visual assessment of the segmentation results

Here, we demonstrate visual examples from the segmentation results to further compare our proposed approach with the baseline models.

### MILDNet is more reliable to outline ROIs

**Figure 9:**
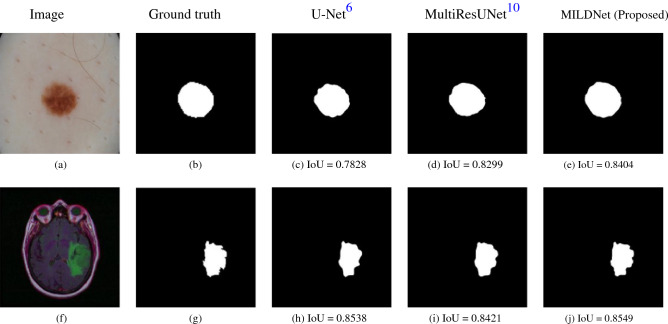
Segmenting a dermoscopy^11,12^ image **(a)** and an MRI^15^ image **(f)** having well-distinguished background and foreground, with **(b,g)** showing their corresponding ground truth segmentation masks. The classical U-Net **(c,h)**, the MultiResUNet **(d,i)**, and the MILDNet **(e,j)** performed equally well in segmenting the ROIs, close to the ground truths.

MILDNet and the other baseline approaches perform favorably in segmenting the medical images with a clear distinction between the background and the ROIs. Figure 9 illustrates images from the ISIC-2018 dermoscopy (Fig. 9a) and the MRI (Fig. 9f) datasets with their corresponding ground truth masks (Fig. 9b) and (Fig. 9g) showing that in case of a clear distinction between the background and the foreground, the classical U-Net (Fig. 9c,h), the MultiResUNet (Fig. 9d,i), and the MILDNet (Fig. 9e,j) perform visually well to segment the ROIs close to the ground truths, however, MILDNet outperforms the other baselines in terms of the IoU in both images.

### MILDNet performs favorably in images with inconsistent foregrounds

**Figure 10:**
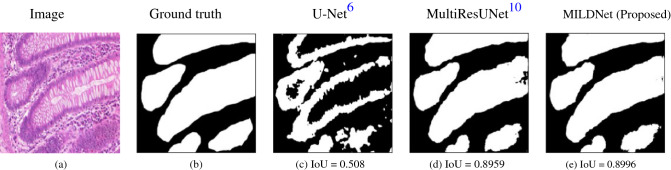
Segmenting a histopathology^16^ image **(a)** and the ground truth mask **(b)**, in which the foreground is not consistent all around. The same staining color intensity and textures in the tumor (ROI) appear also in some non-tumor regions (background). The MILDNet approach **(e)** is consistently better in segmenting this challenging image than the classical U-Net **(c)** and the MultiResUNet **(d)** approaches.

Medical images often contain regions, which appear similar to the background, due to textural and structural similarities, irregularities, and noises. This similarity may lead to loss of information and false negative segmentation. Figure 10a shows a relevant example of such case. Although the ROI boundaries are visually separable between the tumor and the non-tumor regions (see Fig. 10b), the staining color intensity and the textures within the tumor (ROI) and non-tumor (background) appear the same in some regions, providing a challenge for the segmentation. Figure 10c shows that the classical U-Net under-segments the ROIs with IoU of 0.5083 and has missed some information about the consistencies in the foregrounds. The MultiResUNet (Fig. 10d) and the MILDNet (Fig. 10e) perform better than the classical U-Net in preserving the spatial information with IoUs of 0.8959 and 0.8996, respectively. We suggest that the use of MLR blocks allows the MILDNet to preserve the shape and the continuity of the ROIs and hence, reducing the spatial information loss during the segmentation.

### MILDNet segments ROIs with obscure boundaries

**Figure 11:**
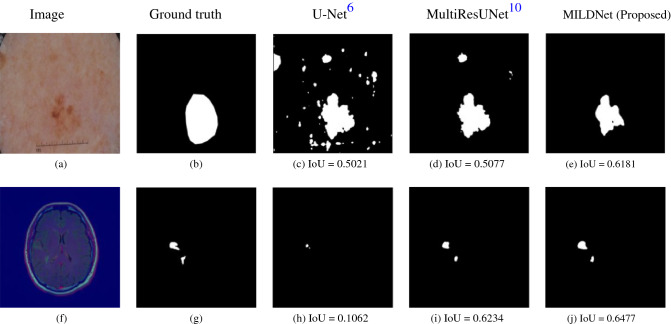
Segmenting a dermoscopy^11,12^ image **(a)** and an MRI^15^ image **(f)** having no clear boundaries separating the foreground and the background, with **(b,g)** demonstrating the ground truth segmentation masks. The classical U-Net either over-segmented **(c)** or under-segmented **(h)** the images, while the MultiResUNet **(d,i)** and the MILDNet **(e,j)** performed considerably better in the segmentation.

Sometimes in the medical images, it is challenging to differentiate the ROIs from the background due to the presence of obscure boundaries. Figure 11a,f illustrate two examples, respectively from the dermoscopy and the MRI images with their corresponding segmentation masks (Fig. 11b) and (Fig. 11g), with no clear separating boundaries. The classical U-Net either over-segmented (Fig. 11c) or under-segmented (Fig. 11h) the ROIs. The MultiResUNet (Fig. 11d,i) and MILDNet (Fig. 11e,j) approaches both performed considerably better than the classical U-Net, however, both models have struggled to properly segment the ground-truths. In both examples, the MILDNet approach achieved a superior segmentation accuracy over the baseline approaches, e.g. the IoU of 0.6181 achieved by MILDNet compared to the IoU of 0.5077 achieved by the MultiResUNet in segmenting the challenging dermoscopy image illustrated in Fig. 11a.

**Figure 12:**
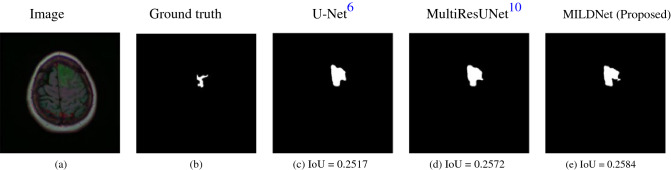
Segmenting a very challenging MRI^15^ image **(a)** having indistinguishable boundaries between the background and the foreground, with **(b)** being the ground-truth. All models including the proposed approach have over-segmented the image **(c–e)**.

Figure 12 further illustrates an extreme case from the MRI dataset (12a) with its ground truth mask (12b), in which the ROI (tumor region) is very difficult to be identified even by a human expert. In this example, all the models (Figs. 12c,d,e) have struggled to properly segment the ROI, resulting in over-segmentation.

### MILDNet is robust against outliers

**Figure 13:**
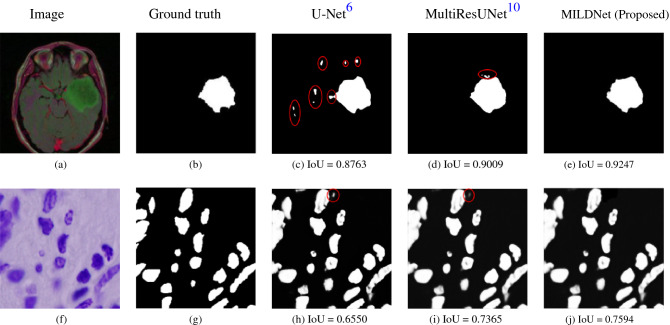
The non-tumor region in the MRI^15^ image **(a)** contains small bright green areas (outliers), which resemble tumor region (ROI). The cell nuclei microscopy^17^ image **(f)** has also some bright particles (outliers), which are visually very similar to the cell nuclei (ROI). MILDNet successfully discarded the outliers from the predicted masks **(e,j)**, with **(b,g)** being the ground truths. Red circles show the incorrectly segmented outliers by the classical U-Net **(c,h)** and the MultiResUNet **(d,i)**.

Segmenting the biomedical images often suffers from outliers, which look very similar to the ROI, but they are not a part of it. Segmentation models often fail to distinguish outliers from the ROIs. Figure 13a illustrates an example from the MRI dataset, in which the non-tumor region contains small light green areas (outliers), which resemble the tumor region (ROI) (Fig. 13b). Similarly, Fig. 13f illustrates another example from the cell nuclei microscopy dataset with a ground truth mask (Fig. 13g), in which the background has some bright particles (outliers), which are very similar to the ROI (cell nuclei). In both examples, the classical U-Net has mistakenly segmented some of the outliers, circled in red in Fig. 13c,h, as being a part of the predicted masks. The MultiResUNet (Fig. 13d,i) performed better than the classical U-Net to discard outliers, however, still mis-classified small background regions. MILDNet (Fig. 13e,j) has successfully discarded those outliers, achieving superior segmentation performance over the classical U-Net and the MultiResUNet, in terms of IoU.

Outliers exist also in other datasets. We have observed that our proposed approach is able to robustly discard the outliers from the predicted masks. The dilated convolutions used in the encoder and the decoder units are likely to contribute towards this success by improving the localization of the ROIs, e.g. the nuclei and the tumor regions, thus, providing more reliable segmentation.

### MILDNet preserves connectivity in boundaries in the majority class

**Figure 14:**
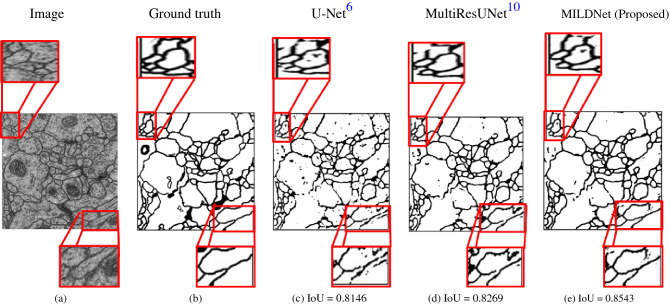
The zoomed areas of the predicted masks in **(c–e)** show that the MILDNet approach can successfully preserves connectivity in boundaries in an electron microscopy^13,14^ image **(a)** with the majority of the class as being the ROI. The ground truth is given in **(b)**.

Usually, ROIs occupy a definite portion of the medical images. The ISBI-2012 electron microscopy dataset provides an interesting segmentation challenge, where the majority of the images contains ROIs (e.g. in Fig. 14a with ground truth mask Fig. 14b). Segmentation models may fail to properly distinguish the foreground and the background in such images, thus, often tend to unnecessarily over-segment the images. Figure 14c shows that the classical U-Net tended to over-segment the ROIs and often missed the spatial information. MultiResUNet (Fig. 14d) and MILDNet (Fig. 14e) both have succeeded to segment the majority of the ROIs, however, MILDNet preserved more contextual information by improving the connectivity between the lines and being more immune to the noises (compare zoomed areas of the predicted masks in Fig. 14c,d,e).

## Conclusion

In this study, we proposed MILDNet, a multi-level dilated residual deep neural network, for the biomedical image segmentation task. We have extended the classical U-Net by (i) incorporating parallel dilated convolutions to extract features from multiple receptive fields to obtain high-level and more detailed features, and (ii) using multi-level residual connections to improve the generalizing capability of the residual learning and to optimize the network during the training process. The proposed approach efficiently captures both the local and the contextual features to segment lesions/tumors by leveraging the inherent properties of the residual learning and the dilated convolutions. We trained and validated the proposed approach on five different biomedical imaging modalities, each with its own segmentation challenges using a 5-fold CV. Our proposed approach consistently outperformed the classical U-Net by relative improvements of 2%, 3%, 6%, 8%, and 14%, respectively for the MRI, the ISIC-2018 dermoscopy, the GlaS-2015 histopathology, the DSB-2018 cell nuclei microscopy, and the ISBI-2012 electron microscopy biomedical images, in terms of DC. MILDNet also outperformed state-of-the-art MultiResUNet approach by relative improvements of 1%, 1%, 1%, 4%, and 4%, respectively for the ISIC-2018 dermoscopy, the DSB-2018 cell nuclei microscopy, the ISBI-2012 electron microscopy, the MRI, and the GlaS-2015 histopathology biomedical images, in terms of DC. Furthermore, the saliency maps showed that MILDNet concentrates much better on the ROIs in biomedical images with complex background.

The visual assessments of the segmentation results further highlighted that the proposed approach improves restoring the spatial and contextual information, i.e. by performing reliably in the presence of outliers and obscure ROI boundaries, and by preserving connectivity in boundaries in the majority class segmentation problem.

We tested our proposed approach as well as the baselines on datasets with different data sizes ranging from 256 in ISBI-2012 and GlaS-2015, to over 2000 in ISIC-2018. We generated image patches to increase the number of samples and applied data augmentation techniques during the training process to avoid over-fitting due to a limited number of data samples in some datasets. The future direction of this study focuses on extending the MILDNet and developing a unified segmentation framework, including 2D and 3D models, for various biomedical imaging modalities and multi-organ semantic segmentation tasks and to further investigate methods to train MILDNet faster with lower memory usage.

## Supplementary Information

Supplementary Information.

## Data Availability

All the imaging data and the corresponding annotations used in this study are publicly available data.

## Code availability

All the source codes and the trained models are open-sourced and freely available online in the manuscript GitHub page at https://gitlab.com/rajgudhe.uef/mildnet.
