# Supplementary material for: Multi-level dilated residual network for biomedical image segmentation
Source: Sci Rep. 2021 Jul 8;11:14105. doi: 10.1038/s41598-021-93169-w (PMC8266898; doi:10.1038/s41598-021-93169-w)
Supplement: Supplementary file 1 — Supplementary Information. [file 41598_2021_93169_MOESM1_ESM.pdf]

Multi-level Dilated Residual Network for Biomedical Image Segmentation

Naga Raju Gudhe[1,*], Hamid Behravan[1,*], Mazen Sudah[2], Hidemi Okuma[2], Ritva Vanninen[2,3], Veli-Matti Kosma[1,4,†], and Arto Mannermaa[1,4,†]

[1]Institute of Clinical Medicine, Pathology and Forensic Medicine, and Translational Cancer Research Area, University of Eastern Finland, P.O. Box 1627,FI-70211, Kuopio, Finland
[2]Department of Clinical Radiology, Kuopio University Hospital, P.O. Box 100, Kuopio,FI-70029, Finland.
[3]Institute of Clinical Medicine, Radiology, and Translational Cancer Research Area, University of Eastern Finland, P.O.Box 1627, FI-70211, Kuopio, Finland.
[4]Biobank of Eastern Finland,Kuopio University Hospital, Kuopio, Finland.
[*]Corresponding first co-authors: raju.gudhe@uef.fi, hamid.behravan@uef.fi
[†]These authors contributed equally to this study.

Supplementary material

Data augmentation

The limited availability of the annotated images is a significant challenge in the domain of medical image analysis. Generating high quality annotations by the expert is also expensive and time consuming. Semantic segmentation of medical images based on deep learning approaches are often prone to overfitting when trained on a limited number of data samples. In this study, we have applied affine, elastic, and pixel-level data augmentation techniques during the training process to address this challenge. The data augmentation generally helps the deep learning models to generalize better on the unseen data (test set).

For the affine transformation, the input image undergoes various operations including, rotation, scaling, flipping and shear. We also used other data augmentation techniques to introduce variations in the pixel intensities by altering brightness, contrast, and adding noise to the original images and also applied deformation operation to alter the geometric shape of the image. We used Albumentations[1], a python library, to apply the multiple transformation techniques. We also performed sanity checks to ensure that medical images and their corresponding ground truth masks undergo similar transformations during the training process. Figure 1 shows some examples of augmented data from DSB-2018[2] dataset.

Figure 2 illustrates learning curves of all datasets used in this study after data augmentation using intersection over union (IoU) as the evaluation metric for each epoch. The learning curves shows that the models are not over-fitting.

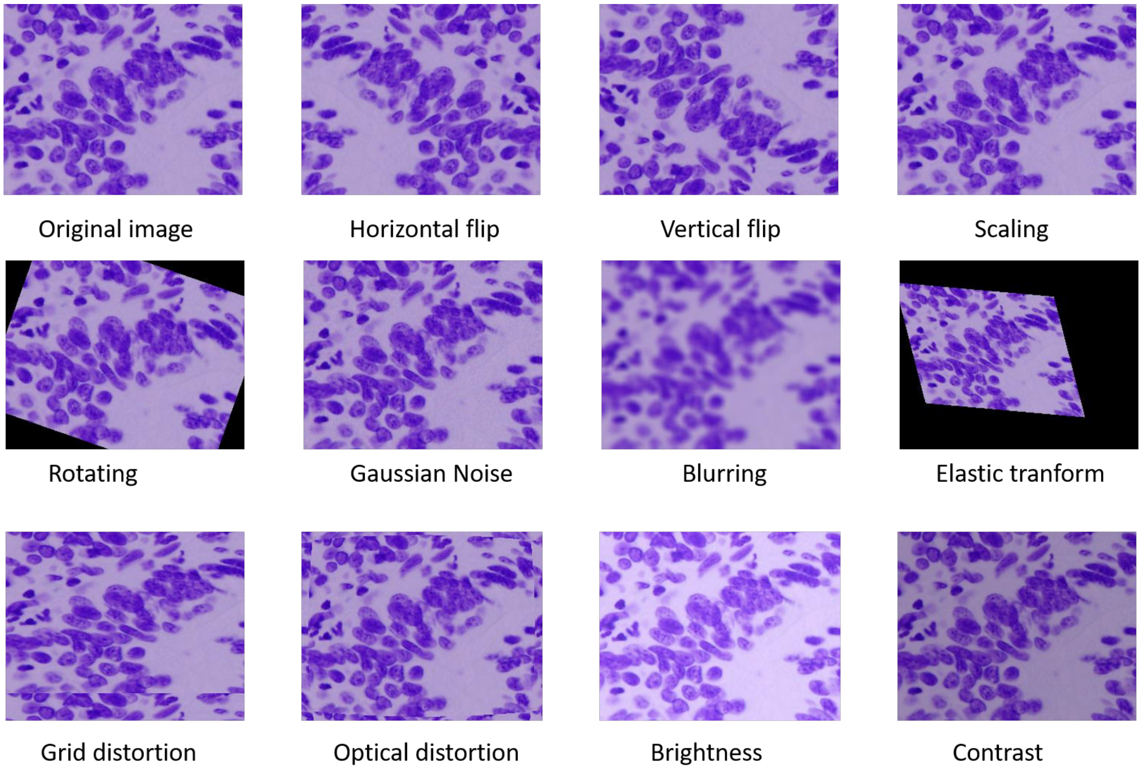

Figure 1. Examples of imaging data generated after applying data augmentation on the DSB-2018 dataset. Transformations are applied during the training process to overcome overfitting.

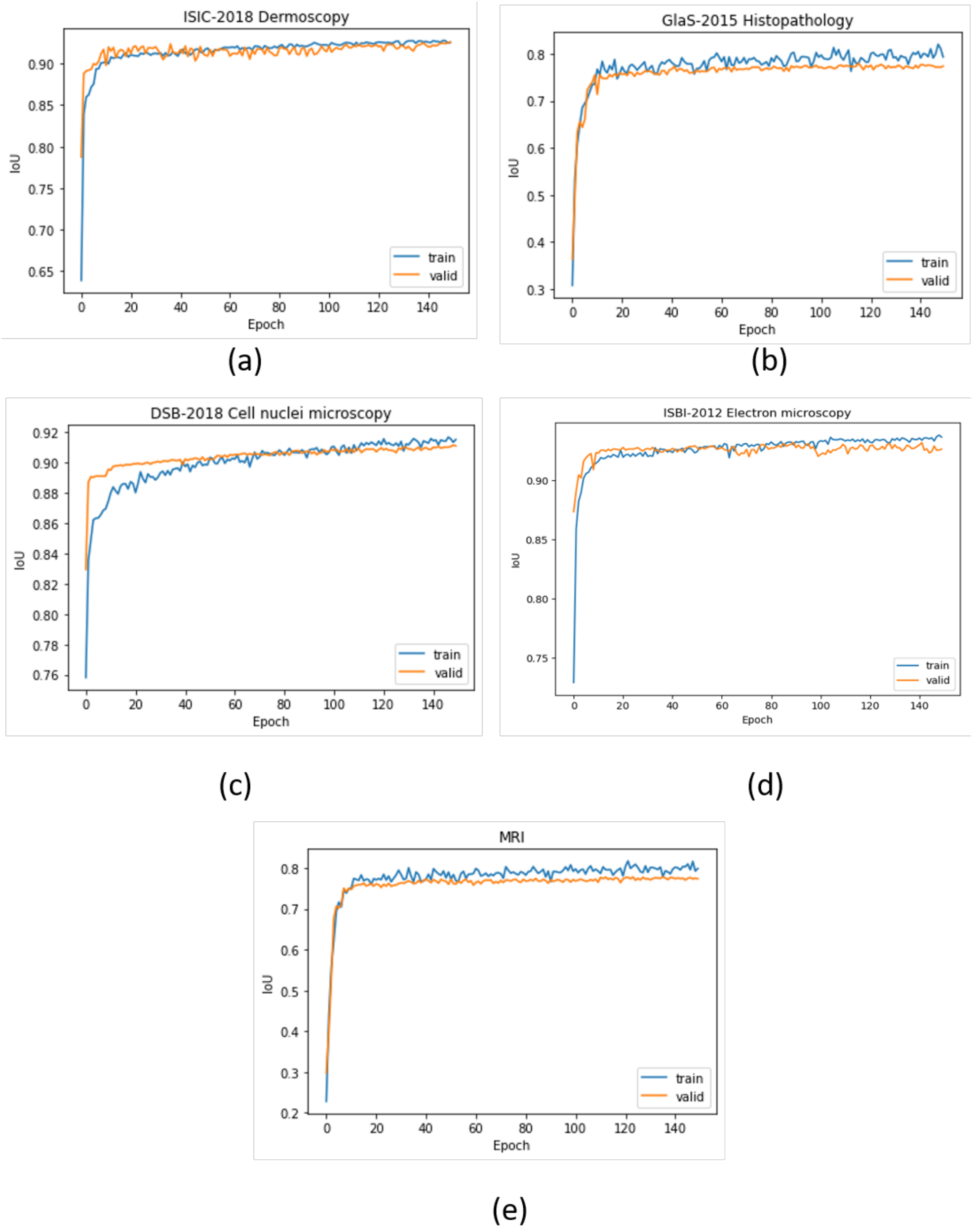

Figure 2. Learning curves for (a) ISIC-2018[3,4], (b) GlaS-2015[5], (c) DSB-2018[2], (d) ISBI-2012[6,7] and (e) MRI[8] datasets show that MILDNet, our proposed approach, does not overfit the training data.

References

1. Buslaev, A. *et al.* Albumentations: fast and flexible image augmentations. *Information* **11**, 125 (2020).

2. Hamilton, B. A. 2018 dataset science bowl: Finding the nuclei in divergent images to advance medical discovery (Accessed 01.06.2021). https://www.kaggle.com/c/data-science-bowl-2018/overview.

3. Codella, N. C. F. *et al.* Skin lesion analysis toward melanoma detection 2018: A challenge hosted by the international skin imaging collaboration (ISIC). *CoRR* **abs/1902.03368** (2019).

4. Tschandl, P., Rosendahl, C. & Kittler, H. The HAM10000 dataset, a large collection of multi-source dermatoscopic images of common pigmented skin lesions. *Sci. Data* **5**, 180161 (2018).

5. Sirinukunwattana, K. *et al.* Gland segmentation in colon histology images: The GlaS challenge contest. *Med. Image Analysis* **35**, 489–502 (2017).

6. Arganda-Carreras, I. *et al.* Crowdsourcing the creation of image segmentation algorithms for connectomics. *Front. Neuroanat.* **9**, 142 (2015).

7. Cardona, A. *et al.* An integrated micro- and macroarchitectural analysis of the Drosophila brain by computer-assisted serial section electron microscopy. *PLoS Biol.* **8**, e1000502 (2010).

8. Buda, M., Saha, A. & Mazurowski, M. A. Association of genomic subtypes of lower-grade gliomas with shape features automatically extracted by a deep learning algorithm. *Comput. Biol. Medicine* **109**, 218–225 (2019).
